# Influences of race/ethnicity in periodontal treatment response and bacterial distribution, a cohort pilot study

**DOI:** 10.3389/froh.2023.1212728

**Published:** 2023-06-12

**Authors:** Bing-Yan Wang, Grayson Burgardt, Kavitha Parthasarathy, Daniel K. Ho, Robin L. Weltman, Gena D. Tribble, Jianming Hong, Stanley Cron, Hua Xie

**Affiliations:** ^1^ School of Dentistry, University of Texas Health Science Center at Houston, Houston, TX, United States; ^2^ Arthur A. Dugoni School of Dentistry, University of the Pacific, San Francisco, CA, United States; ^3^ Department of Clinical Sciences, University of Nevada, Las Vegas, NV, United States; ^4^ School of Nursing, University of Texas Health Science Center at Houston, Houston, TX, United States; ^5^ School of Dentistry, Meharry Medical College, Nashville, TN, United States

**Keywords:** periodontitis, race, treatment response, *Porphyromonas gingivalis*, *Streptococcus cristatus*

## Abstract

**Objectives:**

Periodontitis disproportionately affects different racial and ethnic populations. We have previously reported the higher levels of *Porphyromonas gingivalis* and lower ratios of *Streptococcus cristatus* to *P. gingivalis* may contribute to periodontal health disparities. This prospective cohort study was designed to investigate if ethnic/racial groups responded differently to non-surgical periodontal treatment and if the treatment outcomes correlated to the bacterial distribution in patients with periodontitis before treatment.

**Methods:**

This prospective cohort pilot study was carried out in an academic setting, at the School of Dentistry, University of Texas Health Science Center at Houston. Dental plaque was collected from a total of 75 African Americans, Caucasians and Hispanics periodontitis patients in a 3-year period. Quantitation of *P. gingivalis* and *S. cristatus* was carried out using qPCR. Clinical parameters including probing depths and clinical attachment levels were determined before and after nonsurgical treatment. Data were analyzed using one-way ANOVA, the Kruskal–Wallis test, the paired samples *t*-test and the chi-square test.

**Results:**

The gains in clinical attachment levels after treatment significantly differed amongst the 3 groups–Caucasians responded most favorably, followed by African-Americans, lastly Hispanics, while numbers of *P. gingivalis* were highest in Hispanics, followed by African-Americans, and lowest in Caucasians (*p *= 0.015). However, no statistical differences were found in the numbers of *S. cristatus* amongst the 3 groups.

**Conclusion:**

Differential response to nonsurgical periodontal treatment and distribution of *P. gingivalis* are present in different ethnic/racial groups with periodontitis.

## Introduction

Periodontitis, recently defined as a dysbiotic disease resulting from imbalanced oral microbiota (1), is one of the most widespread inflammatory diseases in adulthood, with an estimated 42% of US dentate adults age 30 years and older suffering from some form of the disease (2). Longitudinal studies on the natural history of periodontitis suggest that modifiable and non-modifiable risk factors potentially influence the onset and progression of the disease (3–9). A series of studies has reported on periodontitis disparity amongst different racial/ethnic groups using the National Health and Nutrition Examination Survey (NHANES) I, II, and III data (7, 10, 11). In each study, African Americans (AA) had a higher incidence of periodontitis than Caucasian Americans (CA) when considering raw data. A doubling of the periodontitis incidence in AA vs. CA has been reported, even after adjusting for all co-factors (11). In a recent analysis (12), the prevalence of periodontitis was found to be highest in Hispanic Americans (HA), followed by AA, and lowest in CA.

Of the more than 700 species detected in the oral cavity only a few have been implicated to be periodontal agents (13–15). One of these putative periodontal pathogens, *P. gingivalis*, has been widely studied. Recently, a keystone pathogen hypothesis relating to the pathogenesis of periodontitis has been proposed, which suggests that the presence of *P. gingivalis* in the oral cavity, even in low-abundance, is capable of disturbing host-microbial homeostasis and thereby inducing periodontitis (16, 17). The distribution of *P. gingivalis* in the periodontal pockets may differ among ethnic/racial groups. Vlachojanni et al. (18) analyzed specific bacteria in subjects with periodontitis ≥40 years old and found out that antibodies against *P. gingivalis* MIX (mixed suspension of ATCC strains 33277 and 53978) for AA were detected 3 times more frequently than that for CA.

The pathogenicity of *P. gingivalis* begins with its bacterial adherence in the oral cavity (19). *P. gingivalis* uses multiple cellular and extracellular components such as fimbriae, proteases, and hemagglutinins for adherence (20, 21). FimA, a major subunit of long fimbriae of *P. gingivalis,* is a well-studied virulence factor contributing to colonization, biofilm formation, cell invasion, bone resorption, and the evasion of host defense systems (22–29). FimA is capable of modifying the host response by activating cytokines such as IL-1, IL-6, IL-8, and TNF-α (21, 30) and also mediates coaggregation of *P. gingivalis* with microbes such as *Actinomyces viscous, Streptococcus gordonii*, and *Streptococcus oralis* (21, 31). Previously, we demonstrated that the expression of *P. gingivalis fimA* was repressed in the presence of arginine deiminase of *Streptococcus cristatus*, which led to inhibition of the formation of *P. gingivalis* biofilms (32–35). The inhibition of biofilm formation by *S. cristatus* arginine deiminase is species-specific and influences *P. gingivalis* only. We also found a negative correlation of distributions of *S. cristatus* and *P. gingivalis* in dental plaques of periodontitis patients and that *S. cristatus* interfered with alveolar bone loss induced by *P. gingivalis* in the murine oral cavity (33, 36).

We have recently demonstrated that increase in levels of *P. gingivalis* and lower ratios of *S. cristatus* to *P. gingivalis* are potential risk factors of disparities in periodontal health and periodontitis severity (37, 38). In this study, we investigated if ethnic/racial groups responded differently to non-surgical periodontal treatment and if the treatment outcomes correlated to the bacterial distribution before treatment. The initial non-surgical treatment for periodontitis is scaling and root planning (SRP). Although SRP has been universally used in the treatment of periodontitis, to our knowledge, it has not been demonstrated if racial/ethnic background influences the response to this treatment. Therefore, this study was designed to test the null hypothesis that racial/ethnic background does not influence the clinical response to SRP and/or the distribution of *P. gingivalis* and *S. cristatus* in periodontitis sites in the oral cavity.

## Materials and methods

### Study design

The study was carried out in an academic setting, at the School of Dentistry, University of Texas Health Science Center at Houston. After screening to determine eligibility and obtaining informed consent, subjects were recruited into the study. All subjects underwent a full-mouth examination for periodontal status, during which microbial samples were collected. Periodontal parameters (probing depth and clinical attachment loss) were abstracted from the Electronic Health Record at our institute, prior to and 6 weeks post nonsurgical periodontal treatment. Participant enrollments and dental plaque sample collections were carried out within a 3-year period. Clinical parameters extraction and statistical analysis were completed after all participants came to their 6-week re-evaluation. Those failed the 6-week follow-up were excluded from our study.

If simple size is calculated based on power analysis using G*power 3.1, by choosing a statistic power = 0.85, *α* = 0.05, and an effect size of 0.2 (small to moderate by Cohen) (39), a total sample size of 279 would be required. However, our study is a pilot study. The study was terminated at 75 participants, when significant differences in treatment response and bacterial distribution amongst 3 racial groups were found.

### Patient enrollment

The research protocol was approved by the Committee for the Protection of Human Subjects of University of Texas Health Science Center at Houston (IRB number: HSC-DB-11-0634). Candidates were screened during their routine dental visits to determine if they meet the inclusion criteria: having been diagnosed as generalized periodontitis Stage II or III, regardless of their grading (40, 41); age of 21–65; and with self-reported ethnicity/race of non-Hispanic Caucasian Americans (CA), non-Hispanic African Americans (AA), or Hispanic Americans (HA). They were excluded from the study if they had antibiotics within 6 months; periodontal therapy within one year; current smokers; pregnant or systemic conditions such as diabetes that are known to influence the outcome of periodontitis treatment. They were enrolled when they met the criteria and signed the written consent for participation. The signed consent forms were stored in a locked drawer in PI's office at the School of Dentistry, UTHealth at Houston. In addition, the participants who failed the 6-week follow-up were excluded from our study.

### Plaque sample collection

Dental plaque samples were collected from mesial or distal sites on two posterior teeth with 5–7 mm probing depth in different quadrants, using paper points before any treatment. The samples contain primarily subgingival dental plaque. The paper points were inserted into the pockets for 1 min and were immersed immediately in 0.5 ml of Tris-EDTA (TE) buffer (pH 7.5). Bacteria were harvested by centrifugation at 16,873×*g* for 3 min. The pellet was resuspended in 50 µl TE buffer. Chromosomal DNA was released by 2 cycles of freezing (at −80°C overnight) and boiling for 20 min.

### Bacterial quantitation by qPCR

*P. gingivalis* cells and *S. cristatus* cells were enumerated by qPCR, using a Bio-Rad CFX 96 real-time PCR system (Bio-Red Laboratories Inc., Redmond, WA, USA). qPCR was performed in duplicate using 5 µl sample DNA, 10 µl SYBR Green PCR mix (Bio-Red Laboratories Inc., Redmond, WA, USA), and 0.4 µM of each forward and reverse primers [TGTAGATGACTGATGGTGAAA and ACTGTTAGCAACTACCGATGT for *P. gingivalis* species-specific *16S rDNA* gene (42) or CTGACGAAGCGAAAGGTCTG and ATGTGGTTGAGCGATACAGC for *S. cristatus arcA* gene], in a total volume of 20 µl. After initial incubation of 95°C for 3 min, denaturation (95°C for 3 s) followed by primer annealing and extension (60°C for 30 s) was performed for 40 cycles, according to manufacturer's recommendation. Standards used to quantitate *P. gingivalis* or *S. cristatus* in the plaque samples were prepared using genomic DNAs from *P. gingivalis* 33277 or *S. cristatus* CC5A (33). The qPCR was performed by individual who was blinded with participant's demographics.

### Non-surgical periodontal treatment

All patients underwent SRP, the standard of care when initiating treatment of periodontitis. SRP was carried out using ultrasonic and hand instruments, under local anesthesia. Five to ten minutes were spent for each tooth, depending on disease severity. SRP was completed in 2 visits. Oral hygiene instruction (brushing and flossing) was given prior to initiating SRP and reinforced at each clinic visit. No other supplementary treatment was provided.

### Determination of clinical parameter

A complete periodontal examination for each patient was carried out before treatment and again 6 weeks after completion of the non-surgical periodontal therapy by the assigned dental student and verified by the supervising clinician. The clinicians participating in the study are board-certified periodontists who had been calibration in measurement of probing depths (PD) and clinical attachment level (CAL) before the initiation of the study.

Two of the parameters, PD and CAL were used for analyzing clinical treatment responses in this study and were abstracted from the Electronic Health Record at our institute. In addition to the clinical treatment outcome measurements at the bacterial sampling sites, the treatment responses of all of the SRP sites (those with ≥5 mm initial PDs) and the full mouth (all teeth excluding wisdom teeth) were analyzed.

### Statistical analysis

One-way analysis of means (ANOVA) for continuous variables and the chi-square test for categorical variables were performed to determine the difference in response to SRP and in bacterial distribution, and patients' demographics. Continuous variables were assessed for normality. Kruskal–Wallis, a non-parametric test, was performed when data was not normally distributed. The comparison of the baseline means vs. the means at the 6-week re-evaluation within each ethnic/racial group was analyzed using the paired samples *t*-test. Linear regression was used to measure the association between the levels of *P. gingivalis* with the treatment responses. A difference was considered significant when a *p*-value <0.05 was obtained.

## Results

### Patient population

Seventy-five subjects diagnosed with generalized periodontitis (based on generalized radiographic alveolar bone loss, >30% sites with CAL >2 mm, and ≥5 mm PD at multiple teeth in ≥2 quadrants) and completed their 6-week follow-up visits were enrolled in the study. Of the total of 75 subjects entered into the study, 17 were AAs, 20 were CAs, and 38 were HAs. The average age of the subjects was 49.4 years, and 49.3 percent of the subjects were women. The subjects had an average of 26.5 teeth. There were no statistical differences in age, gender, or existing numbers of teeth before treatment amongst 3 racial/ethnic groups (Table 1).

**Table 1:** Population demography.

	Subject enrolled	Gender (F/M^a^)	Age	Number of teeth
CA	20	8/12	51.9 ± 10.0	26.75 ± 1.97
AA	17	10/7	50.2 ± 10.7	25.65 ± 4.17
HA	38	19/19	47.8 ± 12.3	26.71 ± 2.85
ALL	75	37/38	49.4 ± 11.4	26.48 ± 3.00

CA, Caucasian Americans; AA, African Americans; HA, Hispanic Americans; ALL, all subject enrolled.

^a^ Female/male.

### Response to SRP

The clinical responses to SRP were analyzed by comparison of CAL and PD before treatment and at 6-week re-evaluation. One-way ANOVA was performed to determine the differences in treatment responses amongst the three ethnic/racial groups, using data from the bacterial sampling sites, the SRP sites, and the full-mouth (the whole dentition excluding wisdom teeth). There were no statistical differences in CALs or PDs at baseline amongst 3 racial/ethnic groups (the Before Treatment columns in Tables 2–4). Analysis of the CAL gains in response to SRP at the two sampling sites revealed a statistically significant difference amongst the 3 groups (*p* = 0.041). *Post hoc* comparisons with the Tukey test indicated that the CAL gains in CAs was statistically different from HAs (*p* = 0.031), but not from AAs (*p* = 0.258). No statistical difference was found between AA and HA groups (*p* = 0.792). No statistical differences in PDs and PD reductions were found between any of the ethnic/racial groups (*p *> 0.05, Table 2).

**Table 2:** Sampling-site clinical parameters in response to SRP*.*

	Before treatment		Re-evaluation		ΔChange	
	CAL	PD	CAL	PD	CAL	PD
CA	5.28 ± 1.83	6.05 ± 1.38	3.58 ± 1.78	4.28 ± 0.96	1.69 ± 1.30	1.83 ± 1.07
AA	4.94 ± 1.52	6.15 ± 0.79	4.00 ± 1.46	4.91 ± 0.92	0.97 ± 0.88	1.19 ± 1.06
HA	4.64 ± 1.67	5.84 ± 1.31	3.93 ± 1.48	4.55 ± 1.41	0.71 ± 1.49	1.26 ± 1.12
ALL	4.88 ± 1.68	5.97 ± 1.22	3.86 ± 1.54	4.56 ± 1.21	1.01 ± 1.37	1.39 ± 1.11
*p*-value	0.398	0.656	0.676	0.325	0.041^a^	0.139

CAL and PD at the bacterial sampling sites are presented as mean ± SD in mm. CA, Caucasian Americans; AA, African Americans; HA, Hispanic Americans; ALL, all subject enrolled.

^a^ The mean difference of ΔChange in sampling-site CAL is significant at the <0.05 level amongst 3 ethnic/racial groups (One-way ANOVA).

**Table 3:** SRP-site clinical parameters in response to SRP*.*

	Before treatment		Re-evaluation		ΔChange	
	CAL	PD	CAL	PD	CAL	PD
CA	4.66 ± 1.28	5.00 ± 0.57	3.84 ± 0.92	3.81 ± 0.66	0.77 ± 1.33	1.19 ± 0.64
AA	4.22 ± 0.92	4.87 ± 0.32	4.46 ± 0.89	3.86 ± 0.51	−0.25 ± 1.35	1.01 ± 0.59
HA	4.39 ± 1.30	4.91 ± 0.49	4.12 ± 0.88	3.75 ± 0.59	0.28 ± 1.33	1.16 ± 0.68
ALL	4.43 ± 1.21	4.93 ± 0.48	4.11 ± 0.91	3.79 ± 0.59	0.30 ± 1.36	1.13 ± 0.64
*p*-Value	0.523	0.666	0.123	0.804	0.077	0.657

CAL and PD at the SRP sites are presented as Mean ± SD in mm. CA, Caucasian Americans; AA, African Americans; HA, Hispanic Americans; ALL, all subject enrolled.

**Table 4:** Full-mouth clinical parameters in response to SRP*.*

	Before treatment		Re-evaluation		ΔChange	
	CAL	PD	CAL	PD	CAL	PD
CA	2.53 ± 0.88	3.08 ± 0.52	2.19 ± 1.01	2.71 ± 0.42	0.37 ± 0.56	0.39 ± 0.35
AA	2.41 ± 0.85	3.34 ± 0.46	2.07 ± 0.83	2.87 ± 0.26	0.35 ± 0.39	0.42 ± 0.32
HA	2.56 ± 0.99	3.19 ± 0.53	2.23 ± 0.37	2.84 ± 0.93	0.27 ± 0.49	0.32 ± 0.29
ALL	2.51 ± 0.92	3.19 ± 0.51	2.18 ± 0.94	2.81 ± 0.36	0.32 ± 0.49	0.36 ± 0.31
*p*-Value	0.872	0.292	0.850	0.348	0.770	0.504

CAL and PD of all sites in the full dentition are presented as Mean ± SD in mm. CA, Caucasian Americans; AA, African Americans; HA, Hispanic Americans; ALL, all subject enrolled.

Analysis of data from the SRP sites revealed similar results as at the bacterial sampling sites. For the CAL gains of all the SRP sites, CA responded most favorably relative to AA and HA, in accordance with the results of the bacterial sampling sites, although the *p* value of one-way ANOVA analysis did not reach significance (*p *= 0.077). There were no statistical differences in the mean PDs and PD reductions amongst three ethnic/racial groups (Table 3).

**Figure 1:**
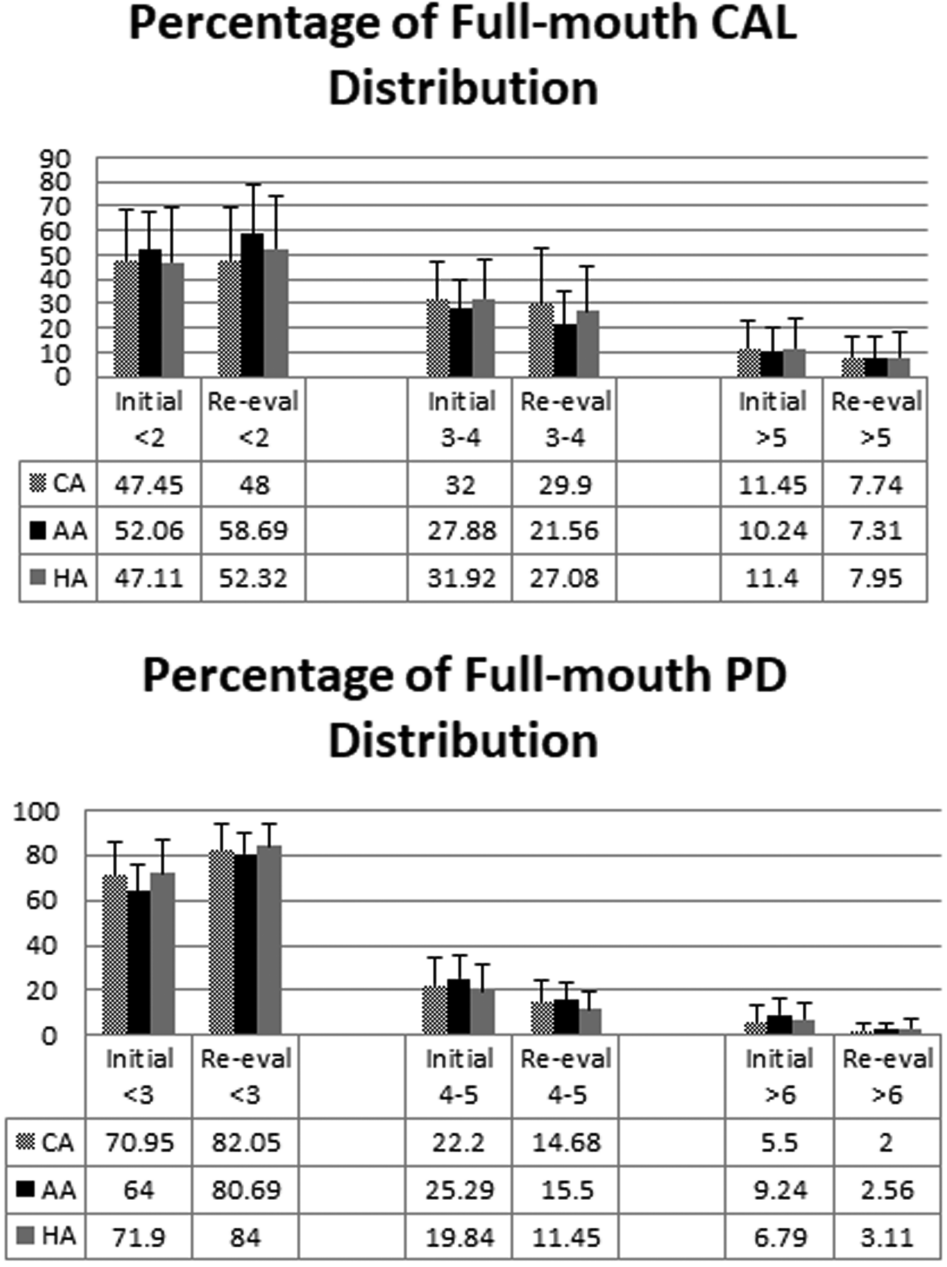
Percentages of full-mouth CALs and PDs (in mm) amongst ethnic/racial groups before and after SRP. CALs and PDs before treatment and at reevaluation were stratified into CAL ≤2 mm, =3–4 mm, ≥5 mm and PD ≤3 mm, =4–5 mm, ≥6 mm. The mean ± SD of percentages of the stratified clinical parameters (CAL on the upper panel, PD on the lower panel) before (Initial) and after (Re-eval) SRP are shown. There was no significant difference amongst 3 ethnic/racial groups (One-way ANOVA).

There was no statistically significant mean difference in any full-mouth clinical parameter amongst 3 ethnic/racial groups (Table 4). Since more than half of the PD sites were ≤3 mm, we analyzed the percentages of full-mouth CAL and PD (in mm) amongst ethic/racial groups before and after SRP. CAL and PD at baseline and re-evaluation were stratified into CAL ≤2 mm, =3–4 mm, ≥5 mm and PD ≤3 mm, =4–5 mm, ≥6 mm. Full-mouth data thus stratified demonstrated an increase in the percentage of sites with PDs of ≤3 mm (from 69.9% to 83.8%) and a decrease in the percentage of sites with PDs of ≥4 mm (from 30.1% to 16.2%) as a result of SRP (Figure 1). Full-mouth data also demonstrate that the percentage of sites with CALs of ≤2 mm increased (from 53.7% to 59.6%) and the percentage of sites with CALs of ≥3 mm decreased (from 46.3% to 39.4%) as a result of SRP. No statistically significant differences were found in PDs, PD reductions, CALs, and CAL gains amongst the three ethnic/racial groups in the full-mouth analysis (Figure 1).

Comparison of the means at the baseline vs. the means at the 6-week re-evaluation within each ethnic/racial group was analyzed using the paired samples *t*-test. There was statistically significant reduction in both PD and CAL at the sample sites, SRP sites and the whole dentition for all the groups (*p* < 0.01).

### Distributions of *P. gingivalis* and *S. cristatus*

Dental plaque samples from the two 5–7 mm PD sites per subject before treatment were used for qPCR to quantitate *P. gingivalis* and *S. cristatus*. As shown in Table 5, HAs exhibited the greatest numbers of *P. gingivalis* and *S. cristatus*, and AAs had more *P. gingivalis* and less *S. cristatus* than CAs. The high skewness values, ranging from 5.249 for *S. cristatus* counts to 5.975 for *P. gingivalis* counts, prevented meaningful comparisons of the mean differences amongst the 3 different ethnic/racial groups. Therefore, Statistically significant disparity in *P. gingivalis* distribution was observed among the three ethnic/racial groups using the Kruskal–Wallis, a nonparametric test (*p* = 0.015). Significant disparity was not found in *S. cristatus* distribution (*p* = 0.630).

**Table 5:** Distribution of *P. gingivalis* and *S. cristatus* in cell counts.

	*P. gingivalis*	*S. cristatus*
CA	3,204 ± 9,914	43,438 ± 117,931
AA	16,615 ± 24,251	18,230 ± 24,564
HA	2,299,608 ± 9,407,308	70,504 ± 2,007,328
*p*-Value	0.015^a^	0.063

Bacterial cell counts were determined by qPCR and presented as Mean ± SD.

^a^ The mean difference of *P. gingivalis* is significant at the 0.05 level amongst 3 ethnic/racial groups (Kruskal–Wallis test).

The correlation of the *P. gingivalis* levels with treatment responses was analyzed using linear regression analysis. Although there were significant differences in the levels of *P. gingivalis* and the changes in CAL after SRP amongst 3 races (Tables 2, 5), association of *P. gingivalis* numbers with either PD or CAL was not detected (*p *= 0.135 and 0.081, respectively). This could be due to the large variation in the levels of *P. gingivalis* and relatively small sample size.

## Discussion

Race has been shown to be one of many risk factors for periodontitis (7, 9, 12, 18). We have previously demonstrated that levels of *P. gingivalis*, a keystone periodontal pathogen, was not evenly distributed among the three racial/ethnic groups, and the ratio of *S. cristatus* to *P. gingivalis* to be significantly higher in CAs than in HAs and AAs (37, 38), which suggest that higher levels of *P. gingivalis* and lower ratios of *S. cristatus* to *P. gingivalis* may contribute to periodontal health disparities.

This study sought to determine if ethnicity/race influences periodontal treatment response and bacterial distribution in dental plaque in periodontitis patients. Disparity in treatment response to SRP and in the distribution of *P. gingivalis* was found among the three ethical/racial groups studied. Therefore, the null hypothesis–racial/ethnic background does not influence response to nonsurgical periodontal therapy and/or the distribution of *P. gingivalis* and *S. cristatus* in periodontal patients, is rejected.

The diagnosis of generalized periodontitis Stage II or III in our inclusion criteria is based on the new classification of periodontitis in the 2017 World Workshop on the Classification of Periodontal and Peri-implant Diseases and Conditions (40, 41). Patients is eligible if they exhibited interdental CAL of 3 mm and above, alveolar bone loss of 15% and beyond, tooth loss due to periodontitis ≤4 teeth. The age limit of 21–65 is to minimize the influence of aging on periodontium. We excluded the antibiotics usage, previous periodontal therapy, current smoker, pregnancy and diabetes, since the usage of antibiotics, periodontal therapy and pregnant will influence the dental biofilm components and/or amount; and current smokers, and diabetes are known to influence the outcome of periodontitis treatment.

Our results indicate that ethnic/racial backgrounds and higher *P. gingivalis* counts may adversely influence the outcome of periodontal treatment. The ethnic/racial backgrounds in this study were based on self-reporting by the subjects after they were diagnosed with generalized periodontitis. Because of the study location (Houston, Texas, USA), majority of the HAs enrolled were Mexican-Americans.

To evaluate response to SRP, we statistically analyzed changes in PDs and CALs, the two most commonly utilized clinical outcome measures. CALs were calculated as PD—(FGM-CEJ) with a negative number of FGM-CEJ (free gingival margin to the cementoenamel junction) indicating gingival recession. In this calculation format, the values of the CALs at reevaluation were less than those before treatment (Tables 2–4). Our calculation format may differ from other institutions and dental offices. The term “gains in CAL” in this article indicated the improvements of CALs in response to SRP, in accordance with other publications (43–46).

The responses to SRP inversely correlate to initial probing depths. Sites with deeper initial probing depths have been found to achieve greater improvements in probing depth reduction and attachment gain after SRP (47). In our study, the dental plaques were sampled from the two 5–7 mm periodontal pockets with an average PD of 5.97 mm before treatment. Since the full-mouth data contained a large percentage of shallow PDs, full-mouth response to treatment was skewed such that the most severely associated sites were masked by the preponderance of shallower sites (Figure 1). Clinical outcome measures for the most severely affected sites were presented separately from the mean whole mouth data to show the response to SRP at those sites exhibiting periodontitis and thus with deep initial probing depths (PDs ≥5 mm). Both the bacterial sampling sites and the SRP-sites (the sites on teeth with PDs measuring ≥5 mm) showed similar treatment outcome, i.e., that CAs responded more favorably to non-surgical periodontal therapy, relative to AAs and HAs (Tables 2, 3).

Total bacterial amounts differed tremendously among the participants of this study with the counts of a specific bacterium varying in a large range as shown in our study (Table 5). This caused an asymmetry of the probability distribution, which was measured by skewness in statistical analysis. Thus, the Kruskal–Wallis test, a nonparametric test, was used instead of ANOVA to analyze bacterial distribution amongst three ethnic/racial groups. Our results indicate that caution should be taken for bacterial count analysis from clinical samples. The standard deviations exceeded the mean counts for each bacterium for every ethnicity/race in our study, indicating a very large range in counts. This skewed distribution in bacterial counts causes shifting of the means towards the few extremely large values, as shown in Table 5 for HAs. Therefore, determination of skewness should be carried out for any parameter, prior to clinical data analysis.

We also analyzed the ratio of *P. gingivalis* or *S. cristatus* to the whole bacterial amount in the plaque, in an attempt to normalize the bacterial distribution. The whole bacterial amount was determined via qPCR using universal primers (*Cyanobacterial 16S rRNA*: GGGCTACACACGYGCWAC, GACGGGCGGTGTGTRCA) (48). However, the percentages of the two bacteria in the total amount exhibited even larger skewness than their original numbers (date not shown), which prohibited us to normalize data in this way.

The treatment responses were analyzed at 6 weeks after SRP in our study. We realize this is much shorter than commonly reported results of 3 months and longer (49–52). However, 6-week re-evaluation is necessary for decision making for further periodontal treatment if deep PDs persist after SRP (53, 54). The current guideline for treating periodontal patient at our School is to re-evaluate 4–6 weeks after SRP. If the deep PDs (≥6 mm) persist, refer the patient for periodontal surgery. Therefore, we chose 6 weeks after SRP as our re-evaluation point. The treatment responses at 6 weeks after SRP in our study were comparable to the 2 reports with the same re-evaluation time points. Statistically significant differences in reduction of CAL and/or PD are detected at 6 weeks after SRP (55, 56). The strength of this study is our emphasis on the racial disparity in treatment response 6 weeks after SRP and in distributions of *P. gingivalis* (a keystone pathogen in periodontitis) and *S. cristatus* (its arginine deiminase inhibits *P. gingivalis* biofilm formation).

This study is carried out in an academic setting. Therefore, the participants may not present the populations in the community. Additionally, the treatment response was evaluated on 75 periodontal patients, only in 6 weeks after SRP. In addition, other periodontal parameters such as bleeding on probing and plaque index are not included. Studies with more participants, longer evaluation period and more clinical parameters will have to be carried out in future to further confirm our findings.

In conclusion, within the limits of this pilot study, disparities exist in clinical response to non-surgical periodontal therapy and in *P. gingivalis* counts amongst ethnic/racial groups with periodontitis, which may merit more frequent periodontal maintenance visits for HAs and AAs after SRP. In addition, caution should be taken for bacterial count analysis from clinical samples, due to the asymmetry of the probability distribution.

## Data Availability

The datasets presented in this article are not readily available because: the original contributions presented in the study are included in the article material. Further inquiries can be directed to the corresponding authors.

## References

[1] Hajishengallis G Lamont RJ. Polymicrobial communities in periodontal disease: their quasi-organismal nature and dialogue with the host. Periodontol 2000. (2021) 86:210–30. 10.1111/prd.12371 33690950 PMC8957750

[2] Eke PI Thornton-Evans GO Wei L Borgnakke WS Dye BA Genco RJ. Periodontitis in US adults: national health and nutrition examination survey 2009–2014. J Am Dent Assoc. (2018) 149:576–88.e6. 10.1016/j.adaj.2018.04.023 29957185 PMC8094373

[3] Genco RJ Borgnakke WS. Risk factors for periodontal disease. Periodontol 2000. (2013) 62:59–94. 10.1111/j.1600-0757.2012.00457.x 23574464

[4] Grossi SG Zambon JJ Ho AW Koch G Dunford RG Machtei EE Assessment of risk for periodontal disease. I. Risk indicators for attachment loss. J Periodontol. (1994) 65:260–7. 10.1902/jop.1994.65.3.260 8164120

[5] Michalowicz BS Aeppli D Virag JG Klump DG Hinrichs JE Segal NL Periodontal findings in adult twins. J Periodontol. (1991) 62:293–9. 10.1902/jop.1991.62.5.293 2072240

[6] Kornman KS Crane A Wang HY di Giovine FS Newman MG Pirk FW The interleukin-1 genotype as a severity factor in adult periodontal disease. J Clin Periodontol. (1997) 24:72–7. 10.1111/j.1600-051X.1997.tb01187.x 9049801

[7] Borrell LN Burt BA Neighbors HW Taylor GW. Social factors and periodontitis in an older population. Am J Public Health. (2004) 94:748–54. 10.2105/AJPH.94.5.748 15117695 PMC1448332

[8] Borrell LN Talih M. Examining periodontal disease disparities among U.S. adults 20 years of age and older: NHANES III (1988–1994) and NHANES 1999–2004. Public Health Rep. (2012) 127:497–506. 10.1177/003335491212700505 22942467 PMC3407849

[9] Thornton-Evans G Eke P Wei L Palmer A Moeti R Hutchins S Periodontitis among adults aged >/=30 years—United States, 2009–2010. Morb Mortal Wkly Rep Surveill Summ. (2013) 62(Suppl 3):129–35. Available at: https://www.cdc.gov/mmwr/preview/mmwrhtml/su6203a21.htm 24264502

[10] Borrell LN Burt BA Gillespie BW Lynch J Neighbors H. Periodontitis in the United States: beyond black and white. J Public Health Dent. (2002) 62:92–101. 10.1111/j.1752-7325.2002.tb03428.x 11989212

[11] Borrell LN Burt BA Warren RC Neighbors HW. The role of individual and neighborhood social factors on periodontitis: the third national health and nutrition examination survey. J Periodontol. (2006) 77:444–53. 10.1902/jop.2006.050158 16512759

[12] Eke PI Dye BA Wei L Slade GD Thornton-Evans GO Borgnakke WS Update on prevalence of periodontitis in adults in the United States: NHANES 2009 to 2012. J Periodontol. (2015) 86:611–22. 10.1902/jop.2015.140520 25688694 PMC4460825

[13] Socransky SS Haffajee AD Cugini MA Smith C Kent RL Jr. Microbial complexes in subgingival plaque. J Clin Periodontol. (1998) 25:134–44. 10.1111/j.1600-051X.1998.tb02419.x 9495612

[14] Jenkinson HF Lamont RJ. Oral microbial communities in sickness and in health. Trends Microbiol. (2005) 13:589–95. 10.1016/j.tim.2005.09.006 16214341

[15] Dewhirst FE. The oral microbiome: critical for understanding oral health and disease. J Calif Dent Assoc. (2016) 44:409–10. 10.1080/19424396.2016.12221033 27514152 PMC7061343

[16] Hajishengallis G Liang S Payne MA Hashim A Jotwani R Eskan MA Low-abundance biofilm species orchestrates inflammatory periodontal disease through the commensal microbiota and complement. Cell Host Microbe. (2011) 10:497–506. 10.1016/j.chom.2011.10.006 22036469 PMC3221781

[17] Hajishengallis G Darveau RP Curtis MA. The keystone-pathogen hypothesis. Nat Rev Microbiol. (2012) 10:717–25. 10.1038/nrmicro2873 22941505 PMC3498498

[18] Vlachojannis C Dye BA Herrera-Abreu M Pikdoken L Lerche-Sehm J Pretzl B Determinants of serum IgG responses to periodontal bacteria in a nationally representative sample of US adults. J Clin Periodontol. (2010) 37:685–96. 10.1111/j.1600-051X.2010.01592.x 20561113

[19] Amano A. Molecular interaction of *Porphyromonas gingivalis* with host cells: implication for the microbial pathogenesis of periodontal disease. J Periodontol. (2003) 74:90–6. 10.1902/jop.2003.74.1.90 12593602

[20] Holt SC Kesavalu L Walker S Genco CA. Virulence factors of *Porphyromonas gingivalis*. Periodontol 2000. (1999) 20:168–238. 10.1111/j.1600-0757.1999.tb00162.x 10522227

[21] Hamada S Amano A Kimura S Nakagawa I Kawabata S Morisaki I. The importance of fimbriae in the virulence and ecology of some oral bacteria. Oral Microbiol Immunol. (1998) 13:129–38. 10.1111/j.1399-302X.1998.tb00724.x 10093527

[22] Belton CM Izutsu KT Goodwin PC Park Y Lamont RJ. Fluorescence image analysis of the association between *Porphyromonas gingivalis* and gingival epithelial cells. Cell Microbiol. (1999) 1:215–23. 10.1046/j.1462-5822.1999.00022.x 11207554

[23] Weinberg A Belton CM Park Y Lamont RJ. Role of fimbriae in *Porphyromonas gingivalis* invasion of gingival epithelial cells. Infect Immun. (1997) 65:313–6. 10.1128/iai.65.1.313-316.1997 8975930 PMC174594

[24] Lamont RJ Chan A Belton CM Izutsu KT Vasel D Weinberg A. *Porphyromonas gingivalis* invasion of gingival epithelial cells. Infect Immun. (1995) 63:3878–85. 10.1128/iai.63.10.3878-3885.1995 7558295 PMC173546

[25] Sandros J Papapanou PN Nannmark U Dahlen G. *Porphyromonas gingivalis* invades human pocket epithelium in vitro. J Periodontal Res. (1994) 29:62–9. 10.1111/j.1600-0765.1994.tb01092.x 8113953

[26] Dorn BR Dunn WA Jr Progulske-Fox A. Invasion of human coronary artery cells by periodontal pathogens. Infect Immun. (1999) 67:5792–8. 10.1128/IAI.67.11.5792-5798.1999 10531230 PMC96956

[27] Dorn BR Burks JN Seifert KN Progulske-Fox A. Invasion of endothelial and epithelial cells by strains of *Porphyromonas gingivalis*. FEMS Microbiol Lett. (2000) 187:139–44. 10.1111/j.1574-6968.2000.tb09150.x 10856647

[28] Deshpande RG Khan MB Genco CA. Invasion of aortic and heart endothelial cells by *Porphyromonas gingivalis*. Infect Immun. (1998) 66:5337–43. 10.1128/IAI.66.11.5337-5343.1998 9784541 PMC108667

[29] Njoroge T Genco RJ Sojar HT Hamada N Genco CA. A role for fimbriae in *Porphyromonas gingivalis* invasion of oral epithelial cells. Infect Immun. (1997) 65:1980–4. 10.1128/iai.65.5.1980-1984.1997 9125593 PMC175257

[30] Lamont RJ Jenkinson HF. Subgingival colonization by *Porphyromonas gingivalis*. Oral Microbiol Immunol. (2000) 15:341–9. 10.1034/j.1399-302x.2000.150601.x 11154429

[31] Yoshimura F Murakami Y Nishikawa K Hasegawa Y Kawaminami S. Surface components of *Porphyromonas gingivalis*. J Periodontal Res. (2009) 44:1–12. 10.1111/j.1600-0765.2008.01135.x 18973529

[32] Xie H Lin X Wang BY Wu J Lamont RJ. Identification of a signalling molecule involved in bacterial intergeneric communication. Microbiology. (2007) 153:3228–34. 10.1099/mic.0.2007/009050-0 17906122 PMC2885614

[33] Wang BY Wu J Lamont RJ Lin X Xie H. Negative correlation of distributions of *Streptococcus cristatus* and *Porphyromonas gingivalis* in subgingival plaque. J Clin Microbiol. (2009) 47:3902–6. 10.1128/JCM.00072-09 19846640 PMC2786655

[34] Xie H Chung WO Park Y Lamont RJ. Regulation of the *Porphyromonas gingivalis* fimA (fimbrillin) gene. Infect Immun. (2000) 68:6574–9. 10.1128/IAI.68.12.6574-6579.2000 11083767 PMC97752

[35] Wu J Xie H. Role of arginine deiminase of *Streptococcus cristatus* in *Porphyromonas gingivalis* colonization. Antimicrob Agents Chemother. (2010) 54:4694–8. 10.1128/AAC.00284-10 20660674 PMC2976165

[36] Xie H Hong J Sharma A Wang BY. *Streptococcus cristatus* ArcA interferes with *Porphyromonas gingivalis* pathogenicity in mice. J Periodontal Res. (2012) 47:578–83. 10.1111/j.1600-0765.2012.01469.x 22448761 PMC4401471

[37] Wang BY Lu T Cai Q Ho MH Sheng S Meng HW Potential microbiological risk factors associated with periodontitis and periodontal health disparities. Front Cell Infect Microbiol. (2021) 11:789919. 10.3389/fcimb.2021.789919 34869082 PMC8637773

[38] Wang BY Cao A Ho MH Wilus D Sheng S Meng HW Identification of microbiological factors associated with periodontal health disparities. Front Cell Infect Microbiol. (2023) 13:1137067. 10.3389/fcimb.2023.1137067 36875522 PMC9978005

[39] Cohen J. A power primer. Psychol Bull. (1992) 112:155–9. 10.1037/0033-2909.112.1.155 19565683

[40] Papapanou PN Sanz M Buduneli N Dietrich T Feres M Fine DH Periodontitis: consensus report of workgroup 2 of the 2017 world workshop on the classification of periodontal and peri-implant diseases and conditions. J Periodontol. (2018) 89(Suppl 1):S173–82. 10.1002/JPER.17-0721 29926951

[41] Tonetti MS Greenwell H Kornman KS. Staging and grading of periodontitis: framework and proposal of a new classification and case definition. J Periodontol. (2018) 89(Suppl 1):S159–72. 10.1002/JPER.18-0006 29926952

[42] Tran SD Rudney JD. Improved multiplex PCR using conserved and species-specific 16S rRNA gene primers for simultaneous detection of *Actinobacillus actinomycetemcomitans*, *Bacteroides forsythus*, and *Porphyromonas gingivalis*. J Clin Microbiol. (1999) 37:3504–8. 10.1128/JCM.37.11.3504-3508.1999 10523542 PMC85679

[43] Haffajee AD Cugini MA Dibart S Smith C Kent RL Jr Socransky SS. The effect of SRP on the clinical and microbiological parameters of periodontal diseases. J Clin Periodontol. (1997) 24:324–34. 10.1111/j.1600-051X.1997.tb00765.x 9178112

[44] Goodson JM Hogan PE Dunham SL. Clinical responses following periodontal treatment by local drug delivery. J Periodontol. (1985) 56(Suppl 11S):81–7. 10.1902/jop.1985.56.11s.81 29538940

[45] Badersten A Nilveus R Egelberg J. Effect of nonsurgical periodontal therapy (VIII). Probing attachment changes related to clinical characteristics. J Clin Periodontol. (1987) 14:425–32. 10.1111/j.1600-051X.1987.tb01548.x 3476520

[46] Mombelli A Muhle T Frigg R. Depth-force patterns of periodontal probing. Attachment-gain in relation to probing force. J Clin Periodontol. (1992) 19:295–300. 10.1111/j.1600-051X.1992.tb00647.x 1517472

[47] Morrison EC Ramfjord SP Hill RW. Short-term effects of initial, nonsurgical periodontal treatment (hygienic phase). J Clin Periodontol. (1980) 7:199–211. 10.1111/j.1600-051X.1980.tb01963.x 7000853

[48] Turner S Pryer KM Miao VP Palmer JD. Investigating deep phylogenetic relationships among cyanobacteria and plastids by small subunit rRNA sequence analysis. J Eukaryot Microbiol. (1999) 46:327–38. 10.1111/j.1550-7408.1999.tb04612.x 10461381

[49] Claffey N Loos B Gantes B Martin M Egelberg J. Probing depth at re-evaluation following initial periodontal therapy to indicate the initial response to treatment. J Clin Periodontol. (1989) 16:229–33. 10.1111/j.1600-051X.1989.tb01646.x 2654197

[50] Aljateeli M Koticha T Bashutski J Sugai JV Braun TM Giannobile WV Surgical periodontal therapy with and without initial scaling and root planing in the management of chronic periodontitis: a randomized clinical trial. J Clin Periodontol. (2014) 41:693–700. 10.1111/jcpe.12259 24730621

[51] Magnusson I Clark WB Low SB Maruniak J Marks RG Walker CB. Effect of non-surgical periodontal therapy combined with adjunctive antibiotics in subjects with “refractory” periodontal disease. (I). Clinical results. J Clin Periodontol. (1989) 16:647–53. 10.1111/j.1600-051X.1989.tb01034.x 2693498

[52] Peikert SA Fischer A Kruse AB Al-Ahmad A Woelber JP Vach K Adjuvant transgingival therapy with visible light plus water-filtered infrared-A (VIS + wIRA) in periodontal therapy-A randomized, controlled, stratified. Double-blinded clinical trial. Antibiotics (Basel). (2021) 10:180–7. 10.3390/antibiotics10030251 33802497 PMC7999319

[53] Cobb CM Sottosanti JS. A re-evaluation of scaling and root planing. J Periodontol. (2021) 92:1370–8. 10.1002/JPER.20-0839 33660307

[54] Claffey N. Decision making in periodontal therapy. The re-evaluation. J Clin Periodontol. (1991) 18:384–9. 10.1111/j.1600-051X.1991.tb02305.x 1890217

[55] Preshaw PM Ide M Bissett SM Holliday R Lansdowne N Pickering K No benefit of an adjunctive phototherapy protocol in treatment of periodontitis: a split-mouth randomized controlled trial. J Clin Periodontol. (2021) 48:1093–102. 10.1111/jcpe.13465 33817809

[56] Schmidt E Kaciroti N Loesche W. Benefits of additional courses of systemic azithromycin in periodontal therapy. Gen Dent. (2011) 59:180–7; quiz 188–9. PMID: . 21903541

